# Synergistic Effects of the Geriatric Nutritional Risk Index and the Modified Creatinine Index for Predicting Mortality in Patients on Hemodialysis

**DOI:** 10.3390/nu14122398

**Published:** 2022-06-09

**Authors:** Takayuki Naito, Toshiki Doi, Kenichi Morii, Koji Usui, Michiko Arita, Kazuomi Yamashita, Kenichiro Shigemoto, Yoshiko Nishizawa, Sonoo Mizuiri, Kensuke Sasaki, Takao Masaki

**Affiliations:** 1 Ichiyokai Yokogawa Clinic, Hiroshima 733-0011, Japan; t.naito@do.enjoy.ne.jp; 2 Department of Nephrology, Ichiyokai Harada Hospital, Hiroshima 731-5134, Japan; k-morii@icy.or.jp (K.M.); k-yamashita@icy.or.jp (K.Y.); ks@icy.or.jp (K.S.); y-nishizawa@icy.or.jp (Y.N.); sm210@med.toho-u.ac.jp (S.M.); 3 Department of Kidney Disease and Community Medicine, Hiroshima University Hospital, Hiroshima 734-8551, Japan; 4 Ichiyokai Clinic, Hiroshima 731-5133, Japan; ku@icy.or.jp; 5 Iciyokai East Clinic, Hiroshima 732-0814, Japan; ma@icy.or.jp; 6 Department of Nephrology, Hiroshima University Hospital, Hiroshima 734-8551, Japan; kensasaki@hiroshima-u.ac.jp (K.S.); masakit@hiroshima-u.ac.jp (T.M.)

**Keywords:** malnutrition, geriatric nutritional risk index (GNRI), modified creatinine index (mCI), hemodialysis, all-cause mortality

## Abstract

This study aimed to investigate whether a combined estimation of the geriatric nutritional risk index (GNRI) and the modified creatinine index (mCI) provides synergistic information for mortality in patients treated by chronic hemodialysis. We analyzed 499 patients on hemodialysis for five years. We set each cut-off value as the high (≥92) and low (<92) GNRI groups and the high (≥21 mg/kg/day) and low (<21 mg/kg/day) mCI groups, and divided them into four subgroups: G1, high GNRI + high mCI; G2, high GNRI + low mCI; G3, low GNRI + high mCI; and G4, low GNRI + low mCI. The survival rate was evaluated and time-to-event analysis was performed. All-cause death occurred in 142 (28%) patients. Kaplan–Meier curves showed that G2 and G4 had a significantly worse outcome (*p* < 0.05) than G1 but not G3. Using the multivariable-adjusted model, only G4 was significantly associated with all-cause mortality compared with G1. Our study suggests that the synergistic effects of the GNRI and the mCI are helpful in predicting all-cause mortality. The combination of these indices may be superior to a single method to distinguish patients who are well or moderately ill from potentially severely ill.

## 1. Introduction

Malnutrition is common, and the nutritional status is strongly associated with adverse outcomes among patients on hemodialysis [1]. To identify malnutrition, several markers or tools have been reported to be useful. The malnutrition-inflammation score (MIS) is a well-established method for assessing the comprehensive nutritional status [2] and is a useful predictor of mortality in this population [3]. Unfortunately, the MIS has lower inter-observer reproducibility than a simple tool. Therefore, the application of the MIS requires subjective evaluations by well-trained examiners.

Dialysis clinicians can calculate the geriatric nutritional risk index (GNRI) by a simple equation using only three nutritional variables, namely serum albumin, height, and body weight [4]. There is a significant inverse correlation between the GNRI and the MIS. The GNRI has the highest accuracy for detecting malnutrition based on the MIS among five screening tools in patients on hemodialysis [5]. A study also showed that the predictability of the GNRI for mortality was similar to that of the MIS in this population [6]. Therefore, the GNRI is widely used in current dialysis practice instead of the MIS.

The creatinine index is used to estimate skeletal muscle mass for patients on hemodialysis [7]. This index is a convenient tool without any device. However, the formula of the original creatinine index was complex and difficult to use in routine assessment. Therefore, the creators of this index produced a simpler version that consisted of age, sex, the pre-dialysis serum creatinine concentration, and single-pool Kt/V for urea, which is termed the “modified creatinine index” (mCI) [8].

Many studies have verified the efficacy of the GNRI for predicting mortality and a meta-analysis showed that a low GNRI was significantly associated with an increased risk of all-cause and cardiovascular mortality in patients treated by chronic hemodialysis [9]. Several studies have also reported that a low mCI score was significantly associated with all-cause, cardiovascular, and infection-related mortality in this population [10,11,12]. Therefore, whether the GNRI or the mCI is preferable has been paid attention. With regard to mortality, a large study showed that GNRI and mCI values were almost equally associated with all-cause and cardiovascular mortality [13]. However, whether these two indices have an equal predictive ability in different cohorts for long follow-ups remains to be clarified. Additionally, studies that focus on the two groups of high GNRI + low mCI and low GNRI + high mCI are required.

Interestingly, two recent studies reported clinical usefulness with combined evaluation of the GNRI and the mCI for mortality in hemodialysis patients [14,15]. However, each study only investigated a relatively small sample size at a single center without any consideration for residual kidney function. The creators of mCI originally developed it for patients without residual kidney function. Therefore, we hypothesized that the association between combination of two indices and mortality would change after adjustment with the presence of residual kidney function. The present study aimed to investigate whether a combined estimation of the GNRI and the mCI provides synergistic information on mortality in patients on hemodialysis using a large sample size at four dialysis facilities, with the inclusion of residual kidney function as one of the covariates.

## 2. Materials and Methods

### 2.1. Study Population

This study was a retrospective, observational study and a post hoc analysis of a multicenter study originally aimed at the validation of biomarkers for long-term mortality in patients on hemodialysis [16]. The entry period spanned from 1 December 2011 to 30 November 2012, and the end of follow-up was November 2017. Inclusion criteria were adult outpatients who received hemodialysis three times each week. We excluded patients who were younger than 20 years, those who underwent combined therapy with peritoneal dialysis, and those who received hemodialysis on a different schedule. Patients who had a poor prognosis for advanced cancer, active infection, severe symptoms of heart failure during rest (New York Heart Association classification IV), or mental health disorders were also excluded. We defined smokers as current smokers and ever smokers, and checked for the presence of residual kidney function by asking the question “Do you still urinate over 100 mL per day?” We enrolled 530 patients from our medical corporation named “Ichiyokai”, which consists of four dialysis facilities. Patients with any missing data regarding baseline characteristics were also excluded from present study. The remaining 499 patients were included in this analysis.

### 2.2. Data Collection

We collected pre-hemodialysis blood samples at the first dialysis session of the week. Samples were examined at a standardized laboratory in Ichiyokai Harada Hospital. We extracted clinical data from electronic medical records and checked survival data every 6 months for 5 years in the original study. The outcome was all-cause mortality. Cardiovascular disease was defined as heart failure, stroke, acute myocardial infarction, peripheral artery disease, rupture of aortic aneurysm, valvular disease, or arrhythmia requiring any treatments.

### 2.3. Calculation of Nutritional Indices and Patient Grouping

We calculated the GNRI using the following formula [4]:

GNRI = [14.89 × serum albumin (g/dL)] + [41.7 × (actual weight/ideal body weight)]

Ideal body weight (kg) = [height (m)]^2^ × 22 (kg/m^2^)

The actual weight/ideal body weight was set to 1, when the patient’s weight was greater than the ideal body weight.

We calculated the mCI using the following formula [8]:

mCI (mg/kg/day) = 16.21 + 1.12 × (0 for women; 1 for men) − 0.06 × age (years) − 0.08 × single-pool Kt/V for urea + 0.009 × 88.4 × pre-hemodialysis creatinine (mg/dL).

### 2.4. Statistical Analysis

Data are expressed as the median (interquartile range) for continuous variables and the number (percentage) for categorical variables. Correlations were tested by Pearson’s correlation coefficient. To compare the four subgroups, we used the Kruskal–Wallis test for continuous variables. When we detected a significant difference among the four subgroups, we performed multiple comparison analysis with the Steel test and designated group 1 as a control. We used the χ^2^ test for the comparison of categorical variables. We set each cut-off value as follows: high (≥92) and low (<92) GNRI groups and high (≥21 mg/kg/day) and low (<21 mg/kg/day) mCI groups. These groups were divided into four subgroups according to a combination of the cut-off values of these indices: group 1 (G1), high GNRI + high mCI; group 2 (G2), high GNRI + low mCI; group 3 (G3), low GNRI + high mCI; and group 4 (G4), low GNRI + low mCI. We evaluated the survival rate by the Kaplan–Meier method and compared rates using the log-rank test. We performed time-to-event analysis using Cox proportional hazards models. After evaluating the crude hazard ratio (HR), we adjusted HRs for covariates. Our selected covariates were age, sex, dialysis vintage, diabetes, a history of cardiovascular disease, smoking status, presence of residual kidney function, type of vascular access, systolic blood pressure, hemoglobin, total cholesterol, corrected serum calcium, phosphate, intact parathyroid hormone, and C-reactive protein. The body mass index, and serum albumin, urea nitrogen and creatinine concentrations were excluded from the covariates. These exclusions were made because the body mass index and serum albumin values were required for estimating the GNRI, and urea clearance and serum creatinine concentrations were required for calculating the mCI. We considered two-sided *p* < 0.05 values as statistically significant and used the statistical software JMP^®^ 15.0.0 (SAS Institute Inc., Cary, NC, USA) for analyses. For counting the number of patients at risk in each group by the Kaplan–Meier method, we used EZR (Saitama Medical Center, Jichi Medical University, Saitama, Japan), which is a graphical user interface for R (The R Foundation for Statistical Computing, Vienna, Austria).

## 3. Results

### 3.1. Baseline Characteristics of the Patients

The clinical characteristics of all 499 patients at the entry are shown in Table 1. The median (interquartile range) age was 65 (56–74) years, 334 (67%) patients were men, and the dialysis vintage value was 64 (29–136) months. A total of 185 (37%) patients had diabetes, 218 (44%) had a history of cardiovascular disease, 253 (51%) were never smokers, and 155 (31%) had residual kidney function. The median body mass index was 22 (19–24) kg/m^2^, single-pool Kt/V for urea was 1.38 (1.25–1.53), serum albumin concentration was 3.7 (3.5–4.0) g/dL, and serum creatinine concentration was 10.8 (8.7–13.0) mg/dL. Median GNRI and mCI values were 95 (90–100) and 21 (19–24) mg/kg/day, respectively. The numbers of patients in low groups were 159 (32%) in GNRI and 225 (45%) in mCI. The numbers of patients in each subgroup were 230 in G1, 110 in G2, 44 in G3, and 115 in G4. There were significant differences in age, male sex, dialysis vintage, presence of diabetes, history of CVD, presence of RKF, BMI, use of P binders, use of statins, hemoglobin, serum albumin, serum urea nitrogen, serum creatinine, serum total cholesterol, corrected serum calcium, serum phosphate, serum intact PTH, and serum C-reactive protein.

### 3.2. Correlation between the GNRI and the mCI

**Figure 1:**
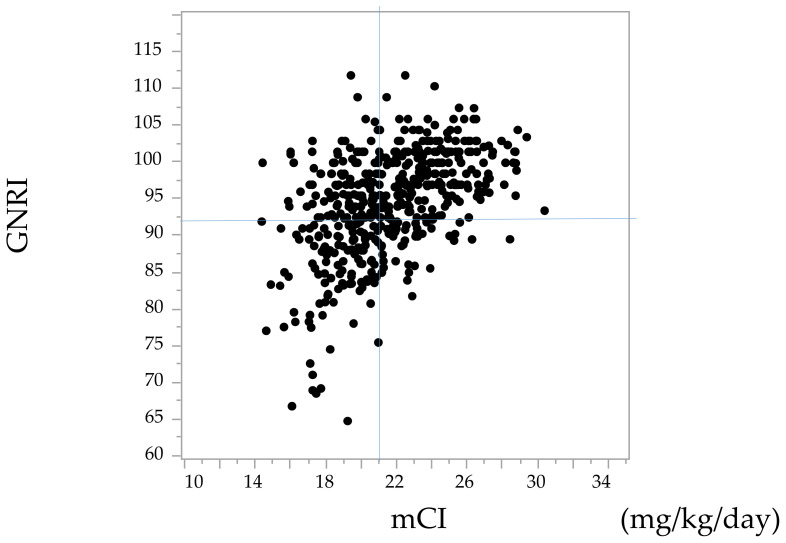
Scatter plot of the geriatric nutritional risk index (GNRI) and the modified creatinine index (mCI). References lines show 92 for the GNRI and 21 mg/kg/day for the mCI.

A scatter plot of the GNRI and the mCI is shown in Figure 1. The correlation coefficient was 0.48, and there was significant correlation between these two indices (*p* < 0.05).

### 3.3. Clinical Outcomes

During a median of 60 (28–60) months of follow-up, 77 censored cases were observed as follows: hospital transfer (*n* = 45), transition to four sessions of hemodialysis each/week (*n* = 14), kidney transplant (*n* = 7), transition to home hemodialysis (*n* = 1), and other reasons (*n* = 10). All-cause death occurred in 142 (29%) patients. Cardiovascular deaths occurred in 64 (12.8%) patients, and these comprised 37 cases of heart failure, 13 cases of stroke, 9 cases of coronary artery disease, 3 cases of aortic aneurysm rupture, and 2 cases of peripheral artery disease. Infection-related deaths occurred in 36 (7.2%) patients, with 24 cases of pneumonia, 9 cases of sepsis, and 2 cases of peritonitis.

**Figure 2:**
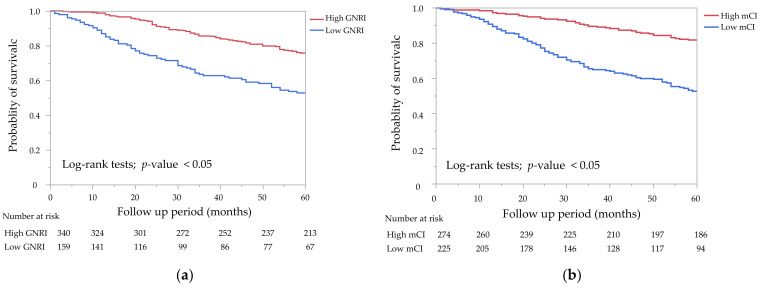
Kaplan–Meier survival curves for all-cause mortality. (**a**) All-cause mortality rates for the two groups of the geriatric nutritional risk index (GNRI) (GNRI < 92 vs. GNRI ≥ 92). The low GNRI group showed a significantly lower survival rate than the high GNRI group (*p* < 0.05). (**b**) All-cause mortality rates for the two groups of the modified creatinine index (mCI) (mCI < 21 mg/kg/day vs. mCI ≥ 21 mg/kg/day). The low mCI group showed a significantly lower survival rate than the high mCI group by the log-rank test (*p* < 0.05).

**Figure 3:**
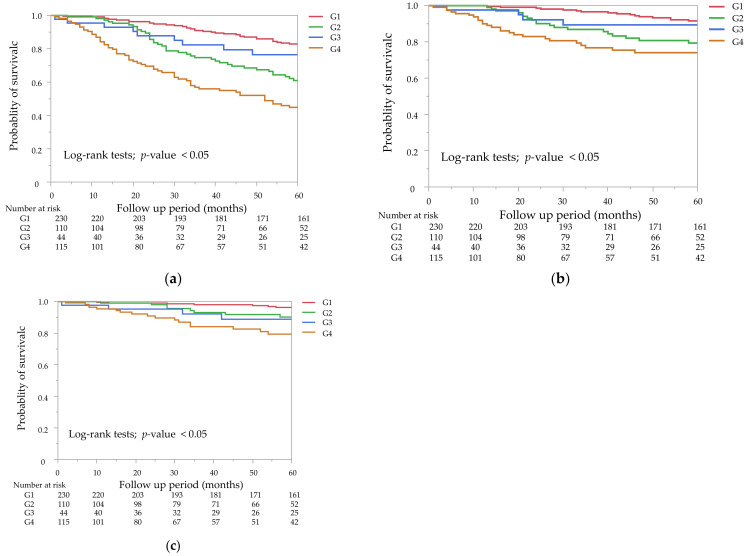
Kaplan–Meier survival curves for all-cause mortality (**a**), cardiovascular mortality (**b**), and infection-related mortality (**c**). Mortality rates for the four subgroups of the combined GNRI and mCI. (**a**) G2 and G4 were significantly different from G1 (*p* < 0.05), but not G3, for all-cause mortality (*p* = 0.23). (**b**) G2 and G4 were significantly different from G1 (*p* < 0.05), but not G3, for cardiovascular mortality (*p* = 0.44). (**c**) G2, G3, and G4 were significantly different from G1 for infection-related mortality (*p* < 0.05).

The Kaplan–Meier curves of the GNRI and the mCI for all-cause mortality showed that the low groups had a significantly lower survival rate than the high groups (Figure 2a,b, *p* < 0.05 by the log-rank test). A combination of the GNRI and the mCI was associated with all-cause, cardiovascular, and infection-related mortality (Figure 3a–c). G2 and G4 showed significantly higher all-cause and cardiovascular mortality compared with G1, but not G3 (*p* = 0.23 for all-cause mortality, *p* = 0.44 for cardiovascular mortality). G2, G3, and G4 showed significantly higher infection-related mortality than G1 (*p* < 0.05).

The unadjusted and multivariable-adjusted HRs by the Cox proportional hazards risk model for all-cause mortality are shown in Table 2. Multivariable-adjusted model 1 was adjusted for age, sex, dialysis vintage, diabetes, a history of cardiovascular disease, smoking status, type of vascular access, systolic blood pressure, hemoglobin, total cholesterol, corrected serum calcium, phosphate, intact parathyroid hormone, and C-reactive protein. Model 2 was adjusted for model 1 plus the presence of residual kidney function. The low GNRI group showed a significantly worse outcome than the high GNRI group in the unadjusted model. Significant differences were also obtained in model 1 and in model 2. Similarly, the low mCI group showed a significantly worse outcome than the high mCI group in all models.

Unadjusted and multivariable-adjusted HRs by the Cox proportional hazards risk model for all-cause mortality, cardiovascular, and infection-related mortality in the four subgroups are shown in Table 3. With regard to all-cause and cardiovascular mortality, G2 and G4 showed a significantly worse outcome than G1 in the unadjusted model. With regard to infection-related mortality, G4 but not G2 showed a significantly worse outcome than G1 in the unadjusted model. The unadjusted HRs for the covariates included in the Cox proportional hazards models were shown in Supplementary Table S1. In model 1 and 2, G4 was significantly associated with all-cause mortality, while G2 was not. In model 2, G4 was significantly associated with a worse outcome for infection-related mortality than G1, while this association was modest for cardiovascular mortality.

## 4. Discussion

In this study, many patients with malnutrition had a low GNRI or mCI. The total number of mixed cases with a high GNRI + low mCI or a low GNR + high mCI was more frequent than that with a low GNRI + low mCI. The high mCI group appeared to show a better survival rate for all-cause mortality than the high GNRI group by Kaplan–Meier curves, particularly from 25 months. Additionally, only the low GNRI + low mCI group was significantly associated with mortality during 5 years of follow-up by the multivariable Cox proportional hazards risk model with the inclusion of residual kidney function. These findings suggest that combined evaluation of these two indices provide synergistic effects on the prediction of mortality compared with a single method.

Several studies have reported that the GNRI and the mCI are associated with all-cause, cardiovascular and infection-related mortality in patients on hemodialysis [7,10,12,17,18]. Although these two nutritional indices are similarly available for predicting adverse events, their potential efficacy may be different. The GNRI is based on serum albumin concentrations, while the mCI is derived from creatinine, which reflects the mass of skeletal muscle. Therefore, the GNRI is a predictor of visceral proteins and mCI is a predictor of somatic proteins. There may be some differences in the degree, timing, or duration of fluctuations between these proteins. The GNRI is also different from the mCI in terms of using body weight as a parameter. In the formula for calculating the GNRI, actual weight/ideal body weight was set to 1 when the actual weight was greater than the ideal body weight. In the high GNRI score group, the effects of overhydration and/or obesity, which contribute to the mortality rate, may be ignored. The effects of overhydration and/or obesity appear to be lower with the mCI than with the GNRI. The mCI can be calculated with the pre-hemodialysis creatinine concentration, which potentially partly adjusts for the effects of overhydration and/or obesity.

Some studies have compared the clinical efficacy with these two indices. A study showed that the GNRI enabled the identification of the severe malnutrition group, but not the normal and moderate malnutrition groups, while the mCI distinguished the normal group from the other groups [19]. With regard to the prognosis, a lower mCI was found to be associated with a higher risk of hospitalization, whereas the GNRI was poorly associated with this risk [20]. These results suggest that there are different properties between these two indices. A recent large study (J-DOPPS) reported that the GNRI and the creatinine index were mostly equal for predicting all-cause and cardiovascular mortality for a median follow-up of 2.2 years [13]. However, more studies are required to compare the abilities between the GNRI and the mCI in different settings, such as other cohorts and different follow-up periods. In our study, the high mCI group appeared to show a better survival rate for all-cause mortality than the high GNRI group by Kaplan–Meier curves, particularly from 25 months. This result indicates that creative use of these different characteristics may provide synergistic information. Studies that focus on whether the mortality risk in the high GNRI + low mCI group is the same or different compared with that in the low GNRI + high mCI group are warranted.

Recently, two studies focused on a combined evaluation of these two indices for evaluating mortality [14,15]. Both of them showed that patients with low GNRI + low mCI scores were strongly associated with a poor outcome. This finding suggested that the combination of these two indices was superior to that of a single method. Yajima et al. analyzed 263 patients on hemodialysis for 3.1 years and clearly showed risk stratification among four subgroups [14]. However, the dialysis vintage of the patients in their study was a median of 1.5 years, which is much shorter than that in our study. Although their study excluded patients who underwent hemodialysis within 6 months, the patients might have had a certain level of residual kidney function. If some patients had heavy proteinuria, the GNRI might have been underestimated. With regard to the mCI, the creators of this index originally developed it for patients without residual kidney function. Unfortunately, their study did not include data on residual kidney function. A benefit of combined evaluation should be validated among patients who have long hemodialysis vintage. Additionally, the outcome of their study was only all-cause mortality. Therefore, cardiovascular and infection-related mortality remain to be clarified. Fujioka et al. investigated 183 patients on hemodialysis with a mean hemodialysis vintage of 97 months for 5.5 years [15]. The hemodialysis vintage and follow-up in their study are similar to those in our study. Their study examined the effect of combined assessments of not only all-cause mortality, but also cardiovascular and infection-related mortality. However, their four subgroups consisted of 32 to 59 patients in each group. There might have been some type I errors present because of the small sample size. Unmeasurable bias or confounders might have been present owing to the single-center nature of their study. Therefore, a larger study is required to clarify reproducibility of their results. Our study was larger than these studies, and we showed that a combined evaluation of two indices was helpful in the prediction of mortality.

Interestingly, one of the studies mentioned above showed that the mortality rate in the low GNRI + high mCI group tended to be higher than that in the high GNRI + low mCI group [14]. This trend was not observed in our study. This discrepancy might be due to three possible reasons. First, even in patients who were categorized as the low GNRI + high mCI group in our study, the median GNRI was 90, which was higher than that in the low GNRI + low mCI group (*p* < 0.05 by the Steel test). Additionally, in the low GNRI + high mCI group, the median value of mCI was 23 mg/kg/day, which was lower than that in the high GNRI + high mCI group. Typical patients, who have both a lower GNRI + a higher mCI, should be required to clarify this issue. Second, the number of patients in the low GNRI + high mCI group might have led to this discrepancy. Although we recruited almost 500 patients, there were only 44 (8.8%) patients in this group. Therefore, our results might have been underpowered. Finally, there might have been inaccuracies in either GNRI or mCI values in the study by Yajima et al. [14] because of interference from residual kidney function.

The high GNRI + low mCI group tended to show a higher mortality rate than that in the low GNRI + high mCI group in our study, which was not observed in the two previous studies mentioned above [14,15]. This discrepancy between studies might have been due to differences in the management of volume control. We only included patients who received hemodialysis three times per week and excluded patients who underwent combined therapy with peritoneal dialysis or received hemodialysis on a different schedule. We also considered a censored case if someone required hemodialysis more frequently than three times per week. These factors might have resulted in the poor prognosis in the high GNRI + low mCI group. Kaplan–Meier curves for cardiovascular mortality showed that the risk in G2 was significantly higher than that in G1. This result suggests that the high GNRI + low mCI group had a higher risk of cardiovascular events than that in the high mCI with high or low GNRI group. If we had merged data with patients who underwent combined therapy with peritoneal dialysis or received hemodialysis more frequently than three times per week, the prognosis in this group might have improved. However, these data did not exist for post hoc study. Although mortality in the high GNRI + low mCI group was not significant by the multivariable-adjusted Cox proportional model, we should not be optimistic about the prognosis in this group.

We categorized the patients into four subgroups as follows: high (≥92) and low (<92) GNRI groups and high (≥21 mg/kg/day) and low (<21 mg/kg/day) mCI groups. Previous studies have shown that a GNRI cut-off value of 91–92 clinically identifies malnourished patients on hemodialysis [5,21]. Therefore, we set the GNRI cut-off value at 92 to divide the patients into two groups. With regard to the mCI, previous studies divided patients into two groups by the cut-off value of 19–22 mg/kg/day [14,15]. They simply set the cut-off value from their median value of the mCI. Although a clinically significant cut-off value of the mCI was not fully established, a previous study showed that patients with an mCI of <21 mg/kg/day showed an increased risk of all-cause mortality and showed a non-linear association by the multivariable-adjusted Cox proportional hazards model with restricted cubic spline regression [13]. In accordance with this previous study, we divided the patients into two groups. If future studies apply different cut-off values, their results may differ from our results.

The strengths of our study are as follows. Firstly, this study had a larger sample size than the previous two studies mentioned above [14,15]. Even when we excluded patients who had severe complications, deaths occurred to some extent, and we examined not only all-cause mortality, but also cardiovascular and infection-related mortality. Secondly, the 5-years of follow-up and the small number of censored cases were also strengths of our study. Thirdly, we included data on the presence of residual kidney function. Finally, although the four dialysis facilities belonged to the same medical corporation, practice patterns in management of anemia, hypertension, setting of dry weight, and dietary intervention were not the same in each facility. These factors may have led to a resemblance of a multicenter study.

Our study has some limitations. First, we only assessed the GNRI and the mCI at baseline. Changes in these indices over time may provide different information. Second, there might have been unmeasured confounders. Third, because we lacked data on the presence of obstructive lung disease, we could not adjust HRs by the Charlson risk index. Therefore, we used smoking status as a covariate, taken together with a history of cardiovascular disease and diabetes. Fourth, although we included data on the presence of residual kidney function, we confirmed it by a simple questionnaire and we did not precisely measure the daily urine volume. Finally, larger scale studies are required to clarify the effect of high GNRI + low mCI or low GNRI + high mCI on all-cause, cardiovascular, and infection-related mortality. Nevertheless, our study highlights the synergistic effects of the GNRI and the mCI.

In conclusion, our study shows that the synergistic effects of the GNRI and the mCI are helpful in predicting all-cause mortality. The combination of these two indices may be superior compared with single use to distinguish patients who are well or moderately ill from potentially severely ill.

## Figures and Tables

**Table 1:** Baseline patient characteristics.

	Total (*N* = 499)	G1 (*N* = 230)	G2 (*N* = 110)	G3 (*N* = 44)	G4 (*N* = 115)	*p*-Value
Age, years	65 (56–74)	58 (49–65)	72 (63–77) *	64 (58–68) *	73 (68–81) *	<0.05
Male sex, *n* (%)	334 (67)	189 (82)	58 (53)	34 (77)	53 (46)	<0.05
Dialysis vintage, months	64 (29–136)	91 (45–154)	38 (16–82) *	93 (45–194)	46 (18–106) *	<0.05
Presence of diabetes, *n* (%)	185 (37)	77 (33)	53 (48)	9 (20)	46 (40)	<0.05
History of CVD, *n* (%)	218 (44)	84 (37)	58 (53)	17 (39)	59 (51)	<0.05
Never smokers, *n* (%)	253 (51)	109 (47)	61 (55)	18 (41)	65 (57)	0.15
Presence of RKF, *n* (%)	155 (31)	58 (25)	46 (42)	7 (16)	44 (38)	<0.05
Type of vascular access—AVF, *n* (%)	464 (93)	218 (95)	102 (93)	43 (98)	101 (88)	0.06
BMI, kg/m^2^	22 (19–24)	23 (21–25)	23 (21–24)	19 (18–21) *	19 (18–21) *	<0.05
Systolic blood pressure, mmHg	155 (139–168)	156 (140–167)	159 (141–172)	150 (136–171)	152 (136–164)	0.07
Single-pool Kt/V	1.38 (1.25–1.53)	1.37 (1.25–1.49)	1.38 (1.23–1.54)	1.39 (1.31–1.54)	1.38 (1.24–1.57)	0.61
Use of Antihypertensive drugs, *n* (%)	370 (74)	176 (77)	82 (75)	31 (70)	81 (70)	0.61
Use of RAS inhibitors, *n* (%)	302 (61)	148 (65)	64 (58)	28 (64)	62 (54)	0.25
Use of ESAs, *n* (%)	445 (89)	200 (87)	101 (92)	38 (86)	106 (92)	0.32
Use of P binders, *n* (%)	407 (82)	214 (93)	75 (68)	37 84)	81 (70)	<0.05
Use of VDRAs, *n* (%)	257 (52)	123 (53)	56 (51)	21 (48)	57 (50)	0.85
Use of statins, *n* (%)	59 (12)	21 (9)	22 (20)	3 (7)	13 (11)	<0.05
Hemoglobin, g/dL	11.0 (10.3–11.8)	11.0 (10.3–11.9)	11.1 (10.5–11.9)	11.2 (10.5–11.8)	10.8 (9.9–11.6) *	<0.05
Serum albumin, g/dL	3.7 (3.5–4.0)	4.0 (3.7–4.1)	3.8 (3.6–4.0) *	3.5 (3.3–3.7) *	3.3 (3.1–3.6) *	<0.05
Serum urea nitrogen, mg/dL	65 (56–74)	69 (62–78)	59 (51–68) *	69 (57–79)	59 (47–70) *	<0.05
Serum creatinine, mg/dL	10.8 (8.7–13.0)	13.0 (11.8–14.5)	8.6 (7.5–9.7) *	12.1 (11.2–12.7) *	8.2 (6.7–9.7) *	<0.05
Serum total cholesterol, mg/dL	153 (132–178)	152 (130–174)	164 (139–183) *	145 (123–174)	151 (133–181)	<0.05
Corrected serum calcium, mg/dL	9.4 (9.0–10.0)	9.5 (9.0–9.9)	9.2 (8.9–9.7) *	9.7 (9.0–10.1)	9.4 (9.0–10.2)	<0.05
Serum phosphate, mg/dL	5.2 (4.5–6.0)	5.5 (4.6–6.1)	5.1 (4.4–5.9)	5.2 (4.4–6.1)	4.7 (4.0–5.7) *	<0.05
Serum intact PTH, pg/mL	113 (48–191)	134 (65–217)	94 (51–182) *	112 (30–169)	86 (28–168) *	<0.05
Serum C-reactive protein, mg/dL	0.09 (0.03–0.29)	0.06 (0.03–0.18)	0.10 (0.04–0.25)	0.12 (0.05–0.40) *	0.19 (0.05–0.78) *	<0.05
GNRI	95 (90–100)	98 (96–101)	97 (94–100) *	90 (86–91) *	86 (83–89) *	<0.05
mCI, mg/kg/day	21 (19–24)	24 (22–26)	19 (18–20) *	23 (22–23) *	19 (18–20) *	<0.05

Abbreviations: CVD: cardiovascular disease, RKF: residual kidney function, AVF: arteriovenous fistula, BMI: body mass index, single-pool Kt/V: single-pool Kt/V for urea, RAS: renin–angiotensin system, ESAs: erythropoiesis stimulating agents, P: phosphate, VDRAs: vitamin D receptor activators, PTH: parathyroid hormone, GNRI: geriatric nutritional risk index, mCI: modified creatinine index. * *p* < 0.05 versus G1.

**Table 2:** Prognostic effect of the geriatric nutritional risk index (GNRI) and the modified creatinine index (mCI).

	Unadjusted Model		Multivariable Model 1		Multivariable Model 2	
	Hazard Ratio (95% CI)	*p*-Value	Hazard Ratio (95% CI)	*p*-Value	Hazard Ratio (95% CI)	*p*-Value
GNRI						
High GNRI	1.00 (reference)	-	1.00 (reference)	-	1.00 (reference)	-
Low GNRI	2.48 (1.78–3.45)	<0.05	1.48 (1.02–2.15)	<0.05	1.51 (1.03–2.20)	<0.05
mCI						
High mCI	1.00 (reference)	-	1.00 (reference)	-	1.00 (reference)	-
Low mCI	3.28 (2.30–4.69)	<0.05	1.67 (1.09–2.58)	<0.05	1.82 (1.17–2.82)	<0.05

Abbreviations: CI, confidence interval.

**Table 3:** Prognostic effects in each subgroup.

	Unadjusted Model		Multivariable Model 1		Multivariable Model 2	
	Hazard Ratio (95% CI)	*p*-Value	Hazard Ratio (95% CI)	*p*-Value	Hazard Ratio (95%CI)	*p*-Value
All-cause mortality						
G1	1.00 (reference)	-	1.00 (reference)	-	1.00 (reference)	-
G2	2.61 (1.65–4.12)	<0.05	1.39 (0.83–2.31)	0.21	1.50 (0.89–2.51)	0.13
G3	1.55 (0.75–3.23)	0.24	1.02 (0.48–2.16)	0.96	1.01 (0.47–2.15)	0.98
G4	4.65 (3.06–7.07)	<0.05	2.10 (1.25–3.52)	<0.05	2.31 (1.36–3.89)	<0.05
Cardiovascular mortality						
G1	1.00 (reference)	-	1.00 (reference)	-	1.00 (reference)	-
G2	2.74 (1.41–5.32)	<0.05	1.45 (0.68–3.02)	0.33	1.62 (0.76–3.46)	0.21
G3	1.49 (0.50–4.47)	0.47	1.00 (0.33–3.80)	1.00	1.00 (0.32–3.07)	0.99
G4	4.20 (2.24–7.88)	<0.05	1.83 (0.85–3.98)	0.12	2.12 (0.96–4.68)	0.06
Infection-related mortality						
G1	1.00 (reference)	-	1.00 (reference)	-	1.00 (reference)	-
G2	2.69 (0.97–7.41)	0.06	1.10 (0.35–3.41)	0.87	1.23 (0.39–3.89)	0.72
G3	3.46 (1.01–11.81)	0.05	2.07 (0.57–7.52)	0.27	2.00 (0.55–7.30)	0.29
G4	6.67 (2.76–16.11)	<0.05	2.67 (0.91–7.77)	0.77	2.95 (1.00–8.70)	<0.05

## Data Availability

The data that support the findings of this study are available from the corresponding author upon reasonable request. The data are not publicly available because of privacy or ethical restrictions.

## Supplementary Materials

The following supporting information can be downloaded at: https://www.mdpi.com/article/10.3390/nu14122398/s1, Table S1. The unadjusted hazard ratio for the covariates included in the Cox proportional hazards models.

## References

[1] Pifer T., McCullough K., Port F., Goodkin D., Maroni B., Held P., Young E. (2002). Mortality risk in hemodialysis patients and changes in nutritional indicators: DOPPS. Kidney Int..

[2] Kalantar-Zadeh K., Kopple J., Block G., Humphreys M. (2001). A malnutrition-inflammation score is correlated with morbidity and mortality in maintenance hemodialysis patients. Am. J. Kidney Dis..

[3] Beberashvili I., Azar A., Sinuani I., Kadoshi H., Shapiro G., Feldman L., Averbukh Z., Weissgarten J. (2013). Comparison analysis of nutritional scores for serial monitoring of nutritional status in hemodialysis patients. Clin. J. Am. Soc. Nephrol..

[4] Bouillanne O., Morineau G., Dupont C., Coulombel I., Vincent J.-P., Nicolis I., Benazeth S., Cynober L., Aussel C. (2005). Geriatric Nutritional Risk Index: A new index for evaluating at-risk elderly medical patients. Am. J. Clin. Nutr..

[5] Yamada K., Furuya R., Takita T., Maruyama Y., Yamaguchi Y., Ohkawa S., Kumagai H. (2008). Simplified nutritional screening tools for patients on maintenance hemodialysis. Am. J. Clin. Nutr..

[6] Chen J., Qin X., Li Y., Yang Y., Yang S., Lu Y., Zhao Y., He Y., Li Y., Lei Z. (2019). Comparison of three nutritional screening tools for predicting mortality in maintenance hemodialysis patients. Nutrition.

[7] Canaud B., Garred L., Argiles A., Flavier J., Bouloux C., Mion C. (1995). Creatinine kinetic modelling: A simple and reliable tool for the assessment of protein nutritional status in haemodialysis patients. Nephrol. Dial. Transplant..

[8] Canaud B., Vallée G.A., Molinari N., Chenine L., Leray-Moragues H., Rodriguez A., Chalabi L., Morena M., Cristol J.-P. (2014). Creatinine index as a surrogate of lean body mass derived from urea Kt/V, pre-dialysis serum levels and anthropometric characteristics of haemodialysis patients. PLoS ONE.

[9] Xiong J., Wang M., Zhang Y., Nie L., He T., Wang Y., Huang Y., Feng B., Zhang J., Zhao J. (2018). Association of Geriatric Nutritional Risk Index with Mortality in Hemodialysis Patients: A Meta-Analysis of Cohort Studies. Kidney Blood Press. Res..

[10] Arase H., Yamada S., Yotsueda R., Taniguchi M., Yoshida H., Tokumoto M., Nakano T., Tsuruya K., Kitazono T. (2018). Modified creatinine index and risk for cardiovascular events and all-cause mortality in patients undergoing hemodialysis: The Q-Cohort study. Atherosclerosis.

[11] Huang C., Lee S., Yang C., Hung S., Chiang C., Huang J., Hung K. (2016). A Simpler Creatinine Index Can Predict Long-Term Survival in Chinese Hemodialysis Patients. PLoS ONE.

[12] Arase H., Yamada S., Hiyamuta H., Taniguchi M., Tokumoto M., Tsuruya K., Nakano T., Kitazono T. (2020). Modified creatinine index and risk for long-term infection-related mortality in hemodialysis patients: Ten-year outcomes of the Q-Cohort Study. Sci. Rep..

[13] Yamada S., Yamamoto S., Fukuma S., Nakano T., Tsuruya K., Inaba M. (2020). Geriatric Nutritional Risk Index (GNRI) and Creatinine Index Equally Predict the Risk of Mortality in Hemodialysis Patients: J-DOPPS. Sci. Rep..

[14] Yajima T., Yajima K., Arao M. (2022). Combined Evaluation of Geriatric Nutritional Risk Index and Modified Creatinine Index for Predicting Mortality in Patients on Hemodialysis. Nutrients.

[15] Fujioka H., Koike T., Imamura T., Tomoda F., Kakeshita K., Yamazaki H., Kinugawa K. (2022). Impact of Geriatric Nutritional Risk Index and Modified Creatinine Index Combination on Mortality in Hemodialysis Patients. Nutrients.

[16] Satoh A., Doi S., Naito T., Nakashima A., Masaki T. (2021). *N*-terminal pro brain natriuretic peptide predicts both all-cause and cardiovascular disease mortality in Japanese hemodialysis patients. Clin. Exp. Nephrol..

[17] Takahashi H., Ito Y., Ishii H., Aoyama T., Kamoi D., Kasuga H., Yasuda K., Maruyama S., Matsuo S., Murohara T. (2014). Geriatric nutritional risk index accurately predicts cardiovascular mortality in incident hemodialysis patients. J. Cardiol..

[18] Matsukuma Y., Tanaka S., Taniguchi M., Nakano T., Masutani K., Hirakata H., Kitazono T., Tsuruya K. (2019). Association of geriatric nutritional risk index with infection-related mortality in patients undergoing hemodialysis: The Q-Cohort Study. Clin. Nutr..

[19] Hwang W., Cho M., Oh J., Lee J., Jeong J., Shin G., Kim H., Park I. (2018). Comparison of creatinine index and geriatric nutritional risk index for nutritional evaluation of patients with hemodialysis. Hemodial. Int..

[20] Suzuki Y., Matsuzawa R., Hoshi K., Koh Y., Yamamoto S., Harada M., Watanabe T., Imamura K., Kamiya K., Yoshida A. (2021). Comparative Analysis of Simplified, Objective Nutrition-Associated Markers in Patients Undergoing Hemodialysis. J. Ren. Nutr..

[21] Panichi V., Cupisti A., Rosati A., Di Giorgio A., Scatena A., Menconi O., Bozzoli L., Bottai A. (2014). Geriatric nutritional risk index is a strong predictor of mortality in hemodialysis patients: Data from the Riscavid cohort. J. Nephrol..

