# Supplementary material for: Synergistic Effects of the Geriatric Nutritional Risk Index and the Modified Creatinine Index for Predicting Mortality in Patients on Hemodialysis
Source: Nutrients. 2022 Jun 9;14(12):2398. doi: 10.3390/nu14122398 (PMC9230177; doi:10.3390/nu14122398)
Supplement: Supplementary file 1 [file nutrients-14-02398-s001.zip › nutrients-1760871-supplementary.pdf]

Table S1. The unadjusted hazard ratio for the covariates included in the Cox proportional hazards models.

	All-cause mortality			Cardiovascular mortality			Infection-related mortality		
	Hazard Ratio	(95% CI)	*p*-Value	Hazard Ratio	(95% CI)	*p*-Value	Hazard Ratio	(95% CI)	*p*-Value
Age, +1 years	1.08	(1.06-1.09)	<0.05	1.08	(1.061.11)	<0.05	1.10	(1.07-1.14)	<0.05
Male sex	0.97	(0.68-1.37)	0.85	0.94	(0.56-1.57)	0.81	1.12	(0.55-2.28)	0.74
Dialysis vintage, +1 months	0.99	(0.99-1.00)	0.21	0.99	(0.99-1.00)	0.68	0.99	(0.99-1.00)	0.36
Presence of diabetes	1.42	(1.02-1.98)	<0.05	1.42	(0.86-2.32)	0.17	1.32	(0.68-2.55)	0.42
History of CVD	1.94	(1.39-2.70)	<0.05	1.92	(1.17-3.16)	<0.05	1.77	(0.92-3.42)	0.09
Never smokers	1.03	(0.74-1.44)	0.83	1.11	(0.68-1.20)	0.66	0.66	(0.34-1.28)	0.22
Presence of RKF	0.79	(0.54-1.14)	0.21	0.69	(0.39-1.22)	0.19	0.60	(0.27-1.31)	0.18
Type of vascular access - AVF	0.97	(0.49-1.91)	0.93	0.98	(0.36-2.71)	0.98	0.90	(0.22-3.74)	0.88
Systolic blood pressure, +10 mmHg	0.93	(0.86-0.99)	<0.05	0.99	(0.89-1.10)	0.85	1.03	(0.89-1.19)	0.70
Hemoglobin, +1 g/dL	0.88	(0.77-1.01)	0.06	0.86	(0.71-1.06)	0.15	0.93	(0.71-1.22)	0.62
Serum total cholesterol, +10 mg/dL	0.99	(0.94-1.04)	0.70	0.96	(0.89-1.03)	0.27	1.01	(0.92-1.11)	0.82
Corrected serum calcium, +1 mg/dL	1.06	(0.85-1.34)	0.59	1.13	(0.80-1.59)	0.48	1.32	(0.84-2.07)	0.23
Serum phosphate, +1 mg/dL	0.78	(0.67-0.90)	<0.05	0.95	(0.77-1.16)	0.61	0.54	(0.40-0.72)	<0.05
Serum intact PTH, +100 pg/mL	0.81	(0.68-0.95)	<0.05	0.84	(0.65-1.05)	0.13	0.84	(0.59-1.12)	0.24
SerumC-reactive protein, +0.1 mg/dL	1.02	(1.01-1.03)	<0.05	1.02	(0.99-1.03)	0.07	1.03	(1.01-1.04)	<0.05

Abbreviations: CVD: cardiovascular disease, RKF: residual kidney function, AVF: arteriovenous fistula, PTH: parathyroid hormone,
